# Present-Focused Behavior as a Rational Adaptation to Precarity

**DOI:** 10.1162/opmi_a_00195

**Published:** 2025-04-02

**Authors:** Arjun Mitra, Narayanan Srinivasan, Nisheeth Srivastava

**Affiliations:** Department of Cognitive Science, Indian Institute of Technology Kanpur and Centre of Behavioral and Cognitive Sciences, University of Allahabad; Departments of Cognitive Science and Computer Science & Engineering, Indian Institute of Technology Kanpur

**Keywords:** time preference, unpredictability, resource shocks, resource scarcity, rational adaptation

## Abstract

Inter-temporal impulsivity has been implicated in several theoretical explanations of the self-reinforcing nature of low socioeconomic status (SES). However, how exactly this interaction transpires is yet to be identified. We hypothesize that impulsivity arises from planning failures due to unpredictable resource demands, and people learn to adapt to this by being present-focused. We tested this hypothesis across three studies using a novel paradigm in which participants used a farming simulator and chose crops with different risk and time preferences. We found that participants’ revealed time preferences adaptively shortened when they faced resource shocks and expanded in the absence of such shocks. We also found greater shrinkage of temporal horizons when these shocks were unpredictable rather than predictable. Our work shows that irrationality need not be invoked to explain the occurrence of present-bias in low SES individuals, and that such behavior may simply be a rational adaptation to the environmental demands of planning under precarity.

## INTRODUCTION

The view that economic precarity leads to behavior that further reinforces poverty has been prominently proposed (Lewis, 1966; Moynihan, 1965), refuted (Ryan, 1976) and, recently, prominently reinstated in social science research (Small et al., 2010; Wilson, 2009, 2012). Since this reintroduction, there has been a blossoming of both humanistic (Hays, 2004; Lamont, 2009) and empirical (Anand & Lea, 2011; Banerjee & Duflo, 2007; Schilbach et al., 2016) investigations aimed at understanding the lived experience of people and communities with low socioeconomic status (henceforth SES in this article). A striking aspect of this new surge of research is the consistent documentation of present-centered behavior among people from low SES communities. This correlation between SES and present focus has been documented through personal surveys (Ludwig et al., 2019), revealed preferences across countries (Dohmen et al., 2016) as well as through theoretical syntheses (Pepper & Nettle, 2017).

Why do people often fail to delay gratification? Life history theories suggest that people growing up poor are cued by the mortality risks like higher crime rates or violent neighborhoods in their environment to prefer sooner rewards than people growing up in resource-rich environments (Griskevicius et al., 2011). It has also been argued that people adjust their preferences based on their ability to ensure their longevity (Daly & Wilson, 2005). Along similar lines, Pepper and Nettle (2017) have proposed an ‘uncontrollable mortality risk’ hypothesis arguing that one will rationally not invest in the future if one frequently faces these extrinsic mortality risks and cannot mitigate them. However, mortality risks have been found to account for only 0.13% of the observed discounting from datasets across 53 countries (Riis-Vestergaard & Haushofer, 2017). Furthermore, mortality risk theories are rarely empirically testable since it is difficult to elicit veridical mortality risk judgments from people in controlled settings. Additionally, while mortality risk may be a possible ‘ultimate’ cause for such behavior, there must be more ‘proximate’ causes that promote impulsivity.

Attempting to provide a proximate explanation, some researchers have proposed a ‘scarcity hypothesis’, i.e., patterns of thought and irrational, impulsive behavior brought about by having to make economic decisions in a resource-scarce environment (Haushofer & Fehr, 2014; Mani et al., 2013; Shah et al., 2012). In this line of research, researchers induce impulsive behavior in the lab by making individuals operate under budgetary constraints. These studies show that under high monetary demands, people with scarce resources tend to over-borrow and save less for the future to mitigate immediate demands. (Shah et al., 2012, 2019). On the other hand, lab-induced precarious conditions like environmental unreliability (Kidd et al., 2013), experiencing negative income shocks (Haushofer & Fehr, 2013) have also been shown to produce present-centric behaviors.

The ‘scarcity hypothesis’ identifies resource scarcity, i.e., functioning in a low-resource environment, as the proximate cause for present-centered behavior. While the experience of resource scarcity is undoubtedly an essential aspect of present-centeredness among individuals in low SES communities, we think the critical element that induces an inability to wait is unforeseen resource demands. Given the intrinsic uncertainty spanning intertemporal choices, we believe people shift to a smaller planning horizon when this uncertainty associated with future plans is exacerbated. Thus, what leads people to plan short-term is not just having low resource budgets but dealing with unexpected environmental demands exhausting those budgets. If this is the case, then future-discounting behavior is exhibited not due to failure in self-control or impatience but as a manifestation of the dynamic interplay of precarious resource demands and preferences across time.

Consistent with our proposal, previous research has demonstrated that negative life experiences like living through the Great Depression can systematically affect people’s financial choices like investing in the stock market (Malmendier, 2021b; Malmendier & Nagel, 2011). Studies also show that people belonging to the low socio-economic communities are more prone to pessimistic bias towards the future (Das et al., 2020). This suggests that personal experiences of idiosyncratic shocks can direct what people view as representative. More recently, Hilbert et al. (2022) have shown that people devalue the future when they encounter household debts instead of savings while managing household finances, and this devaluation vanishes when a positive income spike reduces the debts. Thus, the unpredictability inherent in distal choices can be exacerbated by experiences of erratic shocks making future planning dubious.

The role of unpredictability in changing people’s preferences has also been previously studied in other contexts in the literature - people exposed to harsh, unpredictable conditions act more impulsively and discount the future more (Frankenhuis et al., 2016). Mathematical models have further shown that people’s hyperbolic discounting behavior can be explained by uncertainty in hazard rates (Sozou, 1998). Our proposal offers a unification of these insights, showing how the lived experience of individuals in low SES conditions may adaptively influence their beliefs about how far into the future they can profitably plan, yielding impulsive future-discounting behavior.

### Is Short-Term Planning Rational?

To offer a mechanistic explanation as to why people’s temporal preferences shift, we propose that recurrent, unpredictable resource demands (which we term *resource shocks*) lead to depletion in budgets and, consequently, planning failures for long-term plans. People in low SES communities often deal with low budgets, which tend to deplete faster due to resource shocks often in the form of sudden expenses or loss of income, leading people to abandon their future plans and focus on immediate problems instead. Even high budgetary allocations may deplete when resource shocks are multiple and substantial in nature, leading us to theorize that the frequency and intensity of resource shocks (and not resource budgets) are the critical elements that push people to be impulsive.

**Figure 1.**
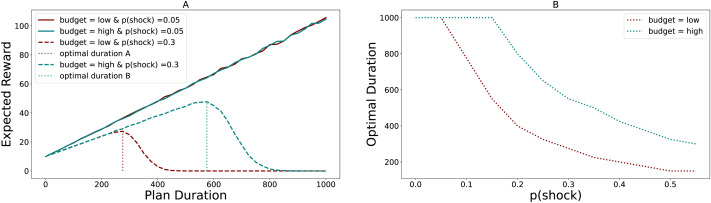
Resource Shock Model - Figure (A) shows how an expected reward of a future plan would look under varied conditions of budgetary allocation and unexpected resource shocks. The expected reward is calculated as the mean reward associated with a plan over 10000 iterations, where the reward is either a positive integer when the future plan is achieved without budget depletion or zero otherwise. Budgetary allocations are either high or low, and resource shocks as frequent or rare. The straight and the dashed lines indicate conditions where resource shocks happen 5% and 30% of the time, respectively. The teal color indicates a high budgetary case, whereas maroon indicates low budgetary conditions. Optimal duration is formalized as the planning duration during which the expected reward of a future plan is maximized under high and low budgets. Optimal duration A and B signifies the planning duration during which the expected reward of a future plan is maximized under frequent resource demands for low and high budgets. Figure (B) shows the change in optimal duration as a function of the probability of resource shock. As the probability of resource demands increases, the optimal planning duration shrinks to a smaller horizon faster when the budget is low.

As an *in silico* presentation of our proposal, we estimate how the expected rewards of future plans and the optimal planning duration (when expected rewards are maximum) change with the plan’s delay to fructification (plan duration). We do this by modulating the frequency of unpredictable resource demands (resource shocks). In this simulation (mathematical details in Supplementary Information), an agent starts a plan to be completed at time *t* = *T* with an initial endowment of resources and a known anticipated reward. At each time step *t* < *T*, one of two things happens: either the endowment is depleted by a resource shock with a probability of 0.05 or 0.30 (i.e., infrequent or frequent), or the reward to be obtained increases as the observer gets closer to time *T*. At time *T*, the simulation ends either with a complete depletion of the observer’s endowment resulting in null payoff or with a positive payoff for waiting. The expected reward is the mean payoff arising from each plan traversal over 10000 iterations^1^. We repeat this process with different initial resource endowments to check if the same results are observed for both low and high endowments. Our simulations reveal some critical insights that we note below:

▪  In cases of infrequent resource shocks (p(shock) = 0.05), we find that the expected reward of a distal plan increases monotonically with plan duration, even under low resource endowments. The same trend was observed for high endowments. This suggests that rational observers should prefer long-term plans when functioning under resource constraints and in the absence of shocks.
▪  When resource shocks become prominent (p(shock) = 0.30), the expected rewards associated with future plans with low resource endowments deplete to zero considerably faster compared to infrequent resource shocks, as shown in Figure 1A. This suggests frequent shocks may deplete low endowments faster, making it infeasible for future goals to be sustained. This could be why people belonging to the low socio-economic community may find long-term plans unsustainable.
▪  Frequent resource shocks also deplete the expected utility of delayed rewards in plans with high endowment. Thus, resource shocks affect long-term planning irrespective of endowment, however, this depletion is less prominent compared to plans with low budgets. This suggests that increased endowment may provide buffer against unexpected expenses.
▪  Optimal duration, operationalized as plan duration where the expected reward of a future plan peaked, changes for both high and low endowments when faced with frequent resource shocks (p(shock) = 0.30). When resources shocks were infrequent, the optimal planning duration for both endowments is equal to the actual planning duration *t* = 1000. However, as resource shocks become more frequent, the optimal planning duration shrink to *t* ≈ 270 and *t* ≈ 570 for low and high budgets, as seen by optimal duration A and B in Figure 1A. Thus, low endowment coupled with frequent, unpredictable turbulence increasingly shortens the planning horizon suggesting these conditions may be necessary and sufficient in pushing people to be present-focused.
▪  Optimal planning duration reduces with increasing frequency of resource shocks for both low and high budgets. However, in the absence of resource shocks (*p* < 0.05), the optimal planning duration remains unchanged at *t* = 1000 even under low endowment, as shown in Figure 1B. Thus, a predictable environment coupled with low endowment might still provide space for individuals to plan for later. However, as unpredictability increases, planning for the future becomes increasingly nonviable for low-budget agents.

Overall, the simulation suggests that frequent resource shocks make longer planning duration unsustainable by draining out resource endowments and minimizing the payoff for waiting. This is prevalent in both high and low endowments, with the minimization amplified when resource endowments are low. However, in the absence of resource shocks, future planning is sustainable even under conditions of low endowment.

Translated back to the context of our theory, waiting for extended periods for rewards while operating under budgetary constraints and frequent resource demands is risky, as plans may fail due to the planner’s inability to commit extra needed resources promptly. This could be interpreted, for instance, as someone failing to pay a credit card bill installment because they lost their job. In such cases, it seems rational for agents to shift their planning horizon to a shorter temporal scale.

### Our Hypothesis

Our proposal resonates with the resource-rationality argument, which proposes that people tend to maximize the expected utility of their choices contingent on their cognitive limitations or ecological constraints (Bhui et al., 2021; Griffiths et al., 2015). The simulation results suggest that the expected utility for waiting is contingent on resource limitations and, more importantly, the recurrence of events that lead to the depletion of said resources. Thus, while waiting for the delayed reward is optimal in the traditional views of rationality, recurrent resource depletions make short-term planning rational in the ‘resource-rational’ (or bounded rationality) perspective.

In this paper, we empirically establish how people’s temporal planning horizons change when subjected to a precarious environment. Our formulation of a precarious environment is motivated by the lived experiences of impoverished individuals. Thus, instead of testing the interactions between resource scarcity and unpredictable shocks, we check if people’s temporal preferences shift under precarious conditions concocted with low endowments and frequent resource shocks. Based on the simulation, we hypothesize that people would adaptively prefer shorter-term plans when they experience unpredictable resource shocks while functioning under a low budget. We also hypothesize that they would prefer long-term goals in the absence of these shocks, i.e., people preferentially choose timescales where they can act most effectively—an ecologically rational strategy.

To test our hypotheses, we developed a novel experimental paradigm to characterize the effect of experimentally induced precarity on time preference^2^. Using this paradigm, we conducted three studies: the first experiment tested whether people’s time preference shifted when they were subjected to precarity induced by resource shocks while operating on a limited budget; the second experiment was a ‘pure control’ experiment, which tested whether people would prefer long-term plans in the absence of such resource shocks, while still operating on a limited budget; the third experiment attempted to pinpoint the critical feature of resource shocks that drives the observed shifts in time preferences in our paradigm.

## EXPERIMENT 1

In our first exploratory study, we simulated environmental precarity within a farming game using resource shocks and sought to measure whether experiencing such shocks led participants to prefer options paid out earlier than later.

### Methods

#### Game Mechanics.

**Figure 2.**
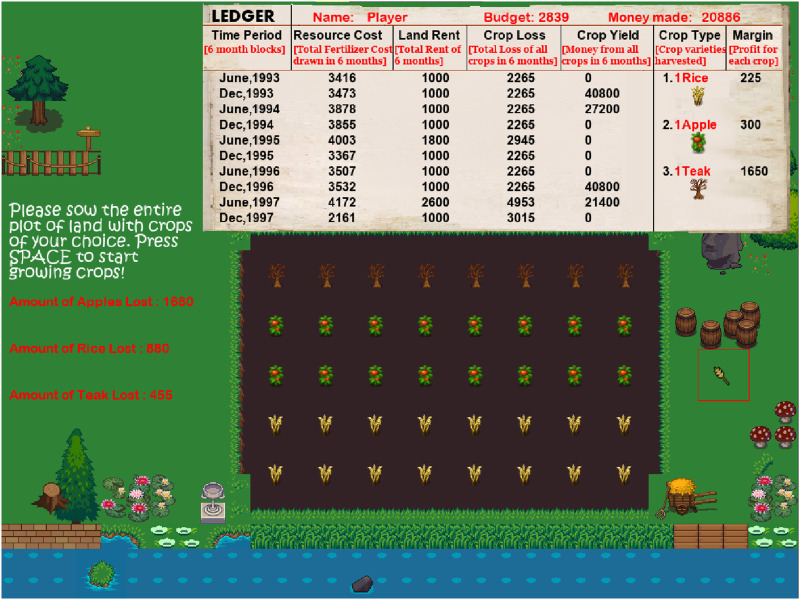
Game GUI: At the top of the user interface is the ‘Ledger’, which displays real-time trial-wise farming-based resource debits and credits. Farming debits included resource debits or farming inputs (‘Resource Cost’), loss of crops (‘Crop Loss’), and rent for the farming land (‘Land Rent’). Farming-based credits included ‘Crop Yield’, i.e., the money earned from harvesting crops. The ledger also displayed each crop variety and the associated profits, total money earned by the player (‘Money Made’), and our formulation of resource scarcity (‘Budget’). Our depiction of environmental precarity was designed as an increased resource cost, crop loss, and land rent in the game. Since participants were encouraged to learn about the risk profile of each crop, we displayed the net loss incurred by each crop after each trial. The middle of the user interface displays the soil where participants could sow and harvest their chosen crops. The participants were informed that crop sowing patterns on the soil or time (month of the year) did not impact crop loss or yield. (Check out https://youtu.be/03nW22OaY7I for a video demonstration of the paradigm.)

Our motivation was to create a close-to-real-life platform where people would experience a precarious environment while planning and exercising their choices through various options, observe their outcomes, and subsequently re-calibrate based on the feedback. Thus, our experimental paradigm was designed as a farming simulator game where participants played the role of a farmer who sowed and harvested crops over multiple trials while observing resource inputs and farming expenses. The GUI shown in Figure 2 details the different design aspects of the game, which is explained in more detail in the Supplementary Information.

##### Farming Game Design.

**Figure 3.**
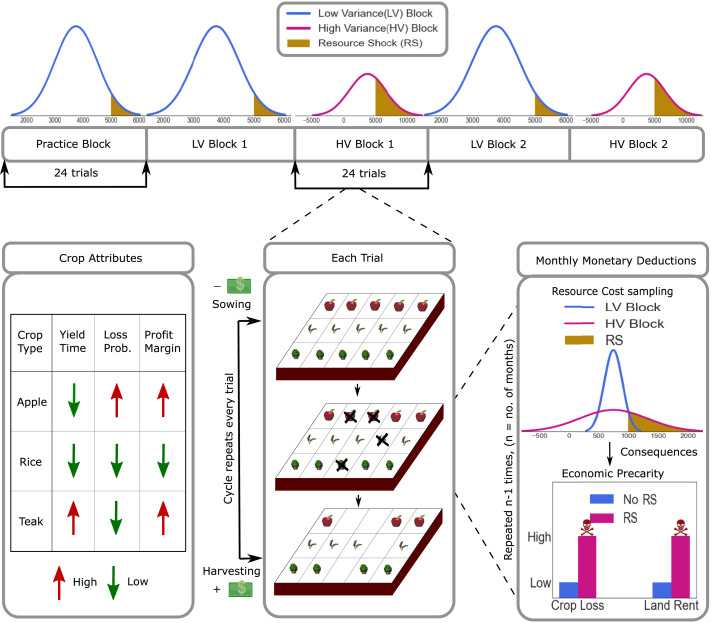
Game Design - this figure depicts the workings of the farming-simulator experimental paradigm. The top panel shows one of the experimental block structures used in the game. The bottom panel shows the varied crop attributes, how every trial traversed through the crop-sowing to the crop-harvesting phase accompanied by crop loss and the critical manipulation of a precarious farming environment.

To simulate a stable and volatile farming environment, we formulated a block design in the game such that farming expenses would be negligible in some blocks (Low Variance or LV) and significant in others (High Variance or HV). These LV and HV blocks were randomly presented to each participant after the presentation of a ‘practice’ block, during which participants got accustomed to the game, as shown in the top panel of Figure 3. Each block had 24 trials, and the game ran for 120 trials. At the start of each trial, participants had to sow crops of their choice on the soil comprising forty slots as shown in the bottom-middle panel, Figure 3. At the end of the trial, they harvested their full-grown crops and started the subsequent trial after resowing the plot. We also made an in-game ledger such that an account of various farming expenses and incomes was readily available enabling participants to make informed crop choices in both LV and HV blocks.

##### Precarity as Resource Shocks.

The game’s central idea was to simulate a resource-scarce environment where farming expenses could go overboard with some probability. Thus, the depiction of environmental precarity and our critical manipulation in this experimental paradigm was the occurrence of resource shocks alongside the formulation of a low budget.

Resource shocks were operationalized as farming expenses going overboard compared to the budget. To be precise, resource shocks were quantified by trials where resource debits in the form of farming expenses (sampled from a random, normal distribution) would rarely go overboard the budget in LV blocks and around one-third of the time in HV blocks. These increased resource debits would manifest as increased crop losses and land rent in those harvest cycles (shown in the bottom-right panel of Figure 3). Thus, in LV blocks, the farming expenses would rarely cross the budget, signifying a smooth, streamlined environment. However, during HV blocks, these expenses would frequently go overboard compared to the budget, suggesting a precarious environment and the occurrence of a resource shock.

##### Crops as Inter-Temporal Choices.

We designed inter-temporal choices in this game as three different crop varieties - Apple, Rice, and Teak - that differed in temporal and risk attributes (displayed on the bottom-left panel of Figure 3). Participants’ choice of crops was our measure of temporal preferences - we wanted to see if people would opt to wait for a more rewarding crop in the face of resource shocks.

In our game, apples and rice were designed as the ‘sooner, smaller reward’ because they took minimal time to grow (yield time) and gave smaller earnings upon harvesting (profit margins). Apple was designed to be a risky choice than rice, i.e., apples would provide higher profits than rice. Still, they were susceptible to higher losses in the face of adversities (loss probability). On the other hand, teak was our depiction of the ‘later, larger reward’ as it had the longest growth time but yielded the most earnings (the effective profit of apple and teak was approximately equal). Thus, teak was the long-term, non-risky choice, apple was the short-term, risky option, and rice was the short-term, non-risky bet. Participants were explicitly informed of the crop yield time and profit margins; however, they were encouraged to estimate the loss probability as they played the game. We hypothesized that participants would show a reduced preference for the long-term crop (teak) after encountering resource shocks.

In our experimental paradigm, participants were asked to sow crops on the entire field so that no slots would be left empty on each trial. Thus, any decrease in long-term crop choices would always be associated with a corresponding increase in short-term choices. However, since we had formulated risky and safe short-term crop choices (apple and rice), we were also interested in the participant’s choice of short-term crops post-shock.

#### Optimal Crop Choice.

**Figure 4.**
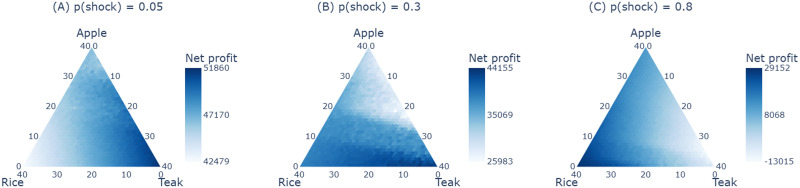
Optimal Crop Choice: These ternary plots show the optimal crop choice under conditions of fixed, low endowment and (A) low, (B) moderate, and (C) high incidences of resource shocks. The teak plantation is the optimal choice in low resource shock conditions. However, as the probability of shock increases, the optimality shifts away from teak. At high probability (p(shock) = 0.8), teak plantation yields a negative net profit—suggesting endowment depletion before harvest is fructified. The values 0 and 40 on the axes denote null and total plantation of those respective crops on the field.

How would an economically optimal agent behave in this game? Would the long-term choice still be optimal under conditions of low endowment and intensifying shock frequencies? To answer these questions, we identified the optimal crop under simulated conditions of a fixed endowment and increasing shock probability. We operationalize optimality in terms of mean net profit (‘Net profit’ on Figure 4) associated with each combination of crops such that ∑(*n*_*a*_, *n*_*r*_, *n*_*t*_) = 40 where *n*_*a*_, *n*_*r*_ and *n*_*t*_ are the number of apple, rice, and teak on plot respectively.

To obtain the optimal crop choice, we derived net profit associated with each possible crop combination under a fixed endowment (*X* = 20000) and low, moderate, and high probability of resource shocks (*p* = 0.05, 0.3 and 0.8). Since opting for teak entitled waiting for teak to reach its full-grown state over six trials, we calculated net profit cumulatively over six trials. On each trial *t* ≤ 6, one of two things happens: our agent can observe a resource shock (determined by *p*), which leads to an increased crop loss and significant endowment depletion, or they encounter no shock leading to a baseline crop expense and a marginal endowment reduction. Thus, on each trial, the endowment is depleted by the crop expenses given by *X*(*t*) = *X*(*t* − 1) − *l*_*i*_ where *l*_*i*_ can be significant or baseline based on the incidence of a shock. If their endowment is completely depleted in this intervening period (i.e., *X*(*t*) ≤ 0 in 1 ≥ *t* < 6), our agent quits the simulation run (or waiting for teak) with the net crop profit accumulated over all preceding *t* trials. Otherwise, they conclude the simulation run at *t* = 6 (i.e., waiting for teak to harvest) with the net profit accrued over all six trials. Net profit (*P*_*net*_) is calculated using the difference between the cumulative capital inflow from crop harvest and capital outflow from crop expenses over *t* trials, which is given by

$$P_{net}=\sum_{1}^{t}\left(p_{i}\right)-\sum_{1}^{t}\left(l_{i}\right)\;\text{where},$$ (1)

$$p_{i}=s_{i}-b_{i}\;\text{and}$$ (2)

$$l_{i}=B\left(1,\theta_{i}\right)*p_{i}*n_{i}$$ (3)

*p*_*i*_ and *l*_*i*_ are the crop yield and crop loss accrued by the *i*_*th*_ crop. Crop yield is obtained from *s*_*i*_ and *b*_*i*_- the selling and buying price associated with the *i*_*th*_ crop. Crop losses *l*_*i*_ are obtained from a binomial distribution determined by loss factor *θ_i_* associated with the *i*_*th*_ crop. *s*_*i*_, *b*_*i*_, and *θ_i_* are based on the crop parameters used in the game. We iterate the above process 1000 times to calculate the mean net profit associated with each crop combination under conditions of a fixed, low endowment and increasing frequency of resource shocks. Figure 4(A), (B), and (C) depict the optimal crop choice under these conditions.

We find that teak plantation leads to highest net profit over time when the probability of shock is low (p(shock) = 0.05). However, as the probability of shock increases by 0.25 (i.e., p(shock) = 0.30), the net profit obtained from the teak plantation shifts away from the maxima. Further increases in shock probability by 0.50 (i.e., p(shock) = 0.80) show that teak plantation produces a negative net profit compared to all other crop combinations. This suggests that waiting for teak to harvest under frequent resource shocks leads to endowment exhaustion before teak is harvested, leading to negative net profit (i.e., a zero crop yield and positive crop loss).

Among the short-term crops, apples provide more net profit than rice as they were designed to provide higher yields than rice under a low probability of shocks (*p* = 0.05). However, as the frequency of shocks increases (*p* = 0.80), apple production increases crop expenses due to its higher-risk profile, making rice production more profitable. Thus, while the short-term, risky choice (apple) is more profitable under low resource shocks, the low-risk, short-term crop (rice) is optimal under frequent shocks.

Overall, these results suggest that the long-term choice is optimal under conditions of minimal turbulence. In other words, teak production pays off when the frequency of shocks is minimal, as arbitrary unexpected expenses do not deplete one’s endowments. However, opting for the long-term choice under incessant turbulence is sub-optimal as heightened expenses may deplete endowments before the future plan is fructified. In such scenarios, proximal, non-risky rewards (similar to rice in our design) pay off immediately, replenishing one’s endowment in the short term. Thus, while teak may be optimal in a streamlined environment, it may not be preferred when resource shocks are frequent.

#### Participants.

Based on pilot data, we predicted a low-to-medium effect of our experimental manipulation. Using G*Power3.1 (Faul et al., 2007), we arrived at a sample size of 72 with power = 0.9, alpha = 0.05, and effect size = 0.35 (using one-tailed one-sample *t*-test). We collected data from 79 participants who were recruited via offline and online adverts on campuses. One participant’s data was discarded as they admitted to having played the game desultorily, and another was discarded for incorrect data records on the part of the game. Eight of these 77 participants were identified as outliers using a 1.75*IQR exclusion criterion on the teak preference shift data (Supplementary analysis including the outliers or robustness checks can be found in Supplementary Information). The analysis was carried forward with 69 participants (30 females and *M*_*age*_ = 25 years).

All participants were recruited with informed consent and monetary compensation. Since the experiment took around sixty to seventy-five minutes, participants were compensated with a standard of $8 (PPP adjusted) for their time. In addition to this, an incentive structure was implemented in the game, where top earners were provided with extra monetary rewards. This was done to nudge people to choose the profit-maximizing crop option in the game. To this effect, three participants were also awarded bonus payments of $25, $15, and $5 (PPP adjusted) based on their performance in the game. The Institutional Ethics Committee approved the study.

### Results

We performed a trial-by-trial analysis to study the effect of resource shocks on time preference based on participants’ choice of crops. We were primarily interested in teak preference, which represents the long-term plan, and its interaction with the short-term plans, i.e., apple and rice preference. Our primary observable variable for this analysis was the number of plants of each variety present on a participant’s field at any point in time, and our primary manipulation of interest was the resource-shock (RS) trials.

#### Long-Term Crop Preference Shifts.

**Figure 5.**
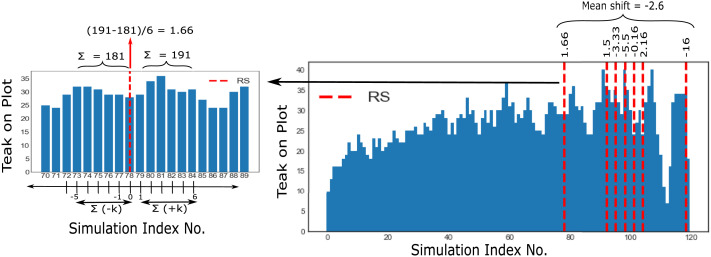
Game Analysis - this figure shows our analysis protocol for the farming-simulator experimental paradigm. For each participant, we identify the trials on which resource shocks occurred, and change in crop preferences is determined by taking the difference in the cumulative amount of crops on k trials before and after each resource shock trial. Here, k is chosen to be six, as teak takes six trials to reach its full-grown state. Each participant’s mean change in crop preference is calculated by taking the mean difference in crop preferences across all resource shocks encountered in the game.

Since the teak harvest could only be fructified after six trials, we focused our analysis on the difference in the cumulative count of teak plants in the field for six trials before and after each resource shock for each participant, as demonstrated in the left panel in Figure 5. The difference in the cumulative teak on the plot across said six trials (Δ*_teak_*) indicated the change in teak preference across each RS trial^3^. We averaged these differences to quantify the mean change in teak preference across all RS trials for every participant ($\delta_{\text{teak}}=\left(\sum_{1}^{m}\Delta_{\text{teak}}\right)/m$ where *m* is the total number of RS trials per participant), as can be seen on the right panel of Figure 5. Finally, the cohort-level change in preference was obtained via the mean change in teak preference across all participants ($\mu_{\delta_{\text{teak}}}=\left(\sum_{1}^{n}\delta_{\text{teak}}\right)/n$ where *n* is the number of participants).

**Figure 6.**
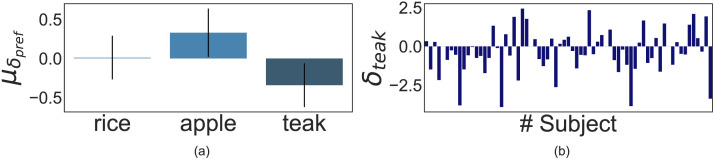
Results of Experiment 1: Panel **(a)** illustrates the mean shift in preference (*μ_δ_pref__*) for all crop choices induced by resource shocks across all participants. The mean shift in rice, apple, and teak preference is 0.01, 0.33, and −0.34, respectively. Error bars signify 95% CI. Panel **(b)** shows the teak preference shift (*δ_teak_*) data for all participants (*n* = 69). Forty participants showed negative teak preference post-resource shock.

A one-sided one-sample *t*-test yielded a significant effect (*M* = −0.34, *SD* = 1.39) of resource shocks on the shift in teak preference (*t*(68) = −2.03, *p* = 0.023, 95% CI [−0.62, −0.06], *d* = 0.24, *BF*_10_ = 1.81). Clearly, as a cohort, our participants reduced their preference for planting teak after having experienced resource shocks, as predicted by our hypothesis. Figure 6(b) plots the mean change in teak preference for all participants, showing that the majority of our participants showed a reduction in teak preference, and the predicted effect also occurred within individuals.

#### Short-Term Crop Preference Shifts.

What do people do instead if they are less likely to plant teak? We found that changes in teak preference were significantly correlated to shifts in both apple and rice preference (*r*(67) = −0.61, *p* < .001, 95% CI [−0.74, −0.44] for apple-teak preference and *r*(67) = −0.49, *p* < .001, 95% CI [−0.65, −0.29] for rice-teak preference). However, a shock-based computation of short-term crop preference shifts elicited that preference for apples significantly increased post resource shocks (*M* = 0.33, *SD* = 1.31, *t*(68) = 2.08, *p* = 0.041, 95% CI [0.01, 0.65], *d* = 0.25, *BF*_10_ = 1) and no preference change for rice (*M* = 0.01, *SD* = 1.18, *t*(68) = 0.08, *p* = 0.933, 95% CI [−0.27, 0.3], *d* = 0.01, *BF*_10_ = 0.13) was found. This suggests that people preferred the short-term risky crop choice more, corresponding to decreases in the long-term crop choice. Our results are summarized in Figure 6(a).

#### Are Preference Shifts Sticky?

Finally, we wanted to see if the effects of resource shocks on the long-term planning horizon, as seen in high variance (HV) blocks, would carry over to a subsequent low variance (LV) block in which people do not experience such shocks. In other words, we wanted to see if preference changes induced by resource shocks were *sticky*. We sought evidence for stickiness in two ways:

▪  Do people show increased teak preference in the LV block immediately encountered after an HV block? If yes, this would suggest evidence for non-stickiness. Furthermore, was there an increase in teak preference in the second LV block compared to the first LV block when both are presented after an HV block? If yes, it would suggest more evidence for non-stickiness.
▪  Do people make more teak in an LV block presented right after an HV block compared to another LV block encountered just before that HV block? If yes, this would suggest that the effects of resource shocks were limited to only precarious HV blocks.

To run this first analysis, we checked if the effect of prior HV blocks showed up in the crop production on subsequent LV blocks (here, we included people whose order of game blocks was HV-HV-LV-LV or HV-LV-LV-HV). We indeed found an increase in mean teak produced in the first LV block compared to the immediate HV block, but it was not statistically significant (*μ*_1*stLV*_ = 27.28, *σ*_1*stLV*_ = 7.97, *μ_priorHV_* = 25.84, *σ_priorHV_* = 5.67, *t*(15) = 1.36, *p* = 0.096, 95% CI [−0.35, 3.23], *d* = 0.20, *BF*_10_ = 1.12). Furthermore, if there were no carryover effects of resource shocks from prior HV to subsequent LV blocks, we would see greater teak preference on the second LV block than the first LV block. Using a paired *t*-test, we found that teak production increases on the second LV block but was not statistically significant (*μ*_1*stLV*_ = 27.28, *σ*_1*stLV*_ = 7.97, *μ*_2*ndLV*_ = 30.18, *σ*_2*ndLV*_ = 10.5, *t*(15) = 1.69, *p* = 0.056, 95% CI [−0.01, 5.81], *d* = 0.3, *BF*_10_ = 1.64). The Bayes’ factors in the analyses tell us that our results show anecdotal evidence for non-stickiness of preference shifts.

Lastly, to further test for non-stickiness, we selected participants who had faced at least one HV block between two LV blocks (i.e., order of blocks was either LV-HV-LV-HV or LV-HV-HV-LV) and compared the mean teak produced on the two LV blocks, excluding the practice block and the last six trials as above. An increase in teak preference in the second LV block compared to the first would suggest non-sticky effect of the intervening HV block onto the subsequent LV block. A one-sided paired *t*-test showed a positive shift in teak preference in the subsequent LV block, but it was not statistically significant (*μ_priorLV_* = 25.73, *σ_priorLV_* = 7.87, *μ_postLV_* = 28.39, *σ_postLV_* = 7.42, *t*(28) = 1.53, *p* = 0.068, 95% CI [−0.24, 5.57], *d* = 0.34, *BF*_10_ = 1.12)—suggesting again anecdotal evidence of non-stickiness of resource shocks in teak production.

### Discussion

Our motivation with this experiment was to simulate an environment that people living in poverty are well versed in. We operationalized this environmental precarity by introducing randomly interspersed resource demands, which went overboard probabilistically. We hypothesized that when people, irrespective of their socioeconomic status, are subjected to such an environment, they naturally shift to a shorter planning horizon. As expected, when faced with economic precarity, designed as resource shocks coupled with crop loss, people did move away from long-term plans (in this context, planting teak in the simulator).

The observed decrease in planting the long-term choice teak was coupled with an increased preference for short-term crops. However, we found that people preferred the risky-sooner bet (apple) more than the safer-sooner option (rice). This is evident from the significant increase in apple preference post-shock and the higher correlation between apple and teak preference shifts compared to rice and teak preference shifts. This implies that our sample was motivated to maintain a high crop-profit margin, and hence, they shifted their preference from a high-profit, low-risk, long-term investment to a high-profit, high-risk, short-term option.

Even though our empirical results point to changes in inter-temporal preference as a function of resource shocks, we think additional mechanisms like decision rules or heuristics may have affected the preference shifts owing to the complexity of the game design. For instance, individuals’ choice of crops may be affected by variety seeking, as we expect them to switch between crop varieties to determine the optimal crop in conditions of null and frequent turbulence. Even though the practice block was designed to minimize such effects, we cannot say with certainty that variety seeking was indeed contained within this block. Furthermore, anchoring effects may have also affected some individuals’ decisions as we find that some individuals show null teak preference shifts (4 people as documented in Figure 6(b)), implying people identified the optimal crop and did not shift from their preferred choice later on.

Now, were these long-term preference shifts sticky? Based on the experience effects literature (Malmendier, 2021a), one may expect that the effects of overruns would be sticky, such that the shortened planning horizon during significant precarity continues to persist in later scenarios of stability. Our results seem to anecdotally suggest that the contracted planning horizon did not carry over to the subsequent blocks in which participants did not face resource shocks. Thus, resource shock-induced time preference changes seem to be transient, at least in this virtual environment.

The discrepancy in our results and previous literature may result from how negative shocks operate in this virtual environment and real-life scenarios. In the gaming paradigm, the manifestation and consequence of a negative shock operated on a time scale of minutes compared to real-life shocks (say, a stock market crash), which can extend over months or years. Furthermore, the ramifications of negative shocks in the game are limited compared to real-life shocks. In our experiments, shocks would result in a transient monetary deficit, which is recoverable in subsequent trials. Real-world shocks of substantial magnitude, often seen in a recession or stock market crashes, may erode one’s wealth, leading to permanent shifts in preference. Thus, the stickiness of preference changes can depend on the time-scales, magnitude or frequency of unpredictable shocks.

## EXPERIMENT 2

Experiment 1 used within-subject manipulation of resource shock frequency to stimulate change in time preferences. However, our model predicted that people would opt for long-term plans in the absence of resource shocks. So, how do people behave when there are no resource shocks? Would they be able to determine the optimal choice? To answer this question, we ran a ‘pure control’ version of the study, wherein participants saw only low-variance blocks of trials, i.e., no resource shocks. Based on the calculations illustrated in Figure 4, we expected an increase in preference for teak over time from participants.

### Methods

#### Design Changes from Experiment 1 to 2.

Since this experiment was designed to ascertain behavior in the absence of resource shocks, we removed the high variance blocks used in the previous experiment. Thus, the game design only consisted of two randomized low-variance blocks after the practice block. The number of trials per block was the same as in Experiment 1. All other methodological designs were kept unchanged.

#### Participants.

We used a Bayesian sample size calculation to determine the number of participants we needed to support the null hypothesis. The null hypothesis for this ‘pure control’ study was that there would be no change in long-term crop (teak) preference across participants (and the alternate hypothesis was a positive change in teak preference). Using SSDttest in R, we arrived at a sample size of 180 participants using a one-sided Bayesian *t*-test with the following specifications: effect size *ρ* = 0.36, power *η* = 0.80, *BF*_*thresh*_ = 3.0 and other default parameters (Fu et al., 2021).^4^ Given this large sample size, we decided to collect data from participants in sets of 20 people. For each set, we also decided the stoppage rule for the data collection to be *BF*_10_ > 3 since *BF*_*thresh*_ was taken to be 3.0 (Fu et al., 2021). Using this criterion, we got the *BF*_10_ for the first set of 20 participants to be 5.68. We found one outlier using a 1.75*IQR exclusion criteria, and proceeded with a final sample size of 19 participants (8 females and *M*_*age*_ = 26 years).

All participants were recruited via an online advert in this experiment as well. Since the experiment took around thirty to forty-five minutes to be completed, participants were compensated with $4 (PPP adjusted) for their time. In addition to this, three participants were also awarded bonus payment of $25, $15 and $5 (PPP adjusted) based on their performance in the game. The institutional ethics committee also approved this study.

### Results

#### Long-Term Crop Preference Shifts.

We conducted the second experiment with participants who did not face any resource shocks, and thus, we devised a strategy for analyzing the data that did not involve shock-based computation of teak preference change. To this end, we calculated the number of teaks on the plot across the LV blocks for every participant and then fitted a linear line to estimate its slope. We hypothesized that we would find a positive slope across participants, i.e., the amount of teak planted would increase within the experiment for each participant. The best fit linear line was calculated based on the teak crop on the plot for all trials - excluding the practice block and the last six trials. The last six trials were excluded based on participants’ feedback that they were motivated not to sow teak on the last six trials as the game would end before harvest (Supplementary analysis that does not involve linear line fitting can be found in Supplementary Information).

**Figure 7.**
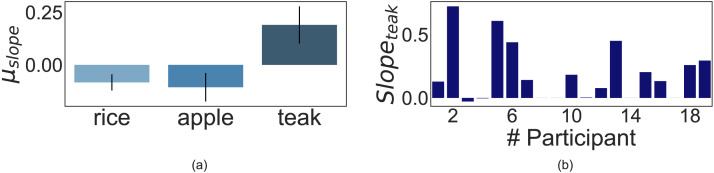
Results of Experiment 2: Panel **(a)** shows the mean slope of the best-fit line to each crop type across the two LV blocks for all participants. Teak showed a significant positive slope, and both Apple and Rice displayed a negative one as the game progressed. Error bars are 95% CIs. Panel **(b)** shows the slope of the best-fit line for only teak across all participants. 13 out of 19 people showed a positive change in teak preference as the game progressed.

Across participants, we calculated the slopes of the best-fit lines as shown in Figure 7(b) and performed *t*-tests. We predicted we would get a positive overall teak preference as participants had not faced any resource shocks. A Bayesian one-sample *t*-test showed substantial evidence in support of the alternate (*BF*_10_ = 50.58). A frequentist one-sided one-sample *t*-test with *H*_0_:*μ*_0_ = 0 and *H*_1_:*μ*_0_ > 0 (where *μ*_0_ = mean slope of overall teak preference across participants) showed that the 95% confidence interval did not include zero (*M* = 0.19, *SD* = 0.22, *t*(18) = 3.725, *p* = .001, 95% CI [0.01, 0.28], *d* = 0.86).

Further investigations into the slope effects of teak preference shifts conducted separately for each LV block revealed a statistically significant positive slope greater than zero in the first LV block (*M* = 0.16, *SD* = 0.39, *t*(18) = 1.80, *p* = .04, 95% CI [0.01, 0.32], *d* = 0.41) and a non-significant positive slope for the second LV block (*M* = 0.19, *SD* = 0.53, *t*(18) = 1.51, *p* = 0.07, 95% CI [−0.02, 0.4], *d* = 0.35). This suggests that most participants had figured out that making teak was the optimal strategy in the first half of the game and continued with it in the absence of shocks.

#### Short-Term Crop Preference Shifts.

We tested similar slope-wise preference changes in apples and rice. Bayesian *t*-tests showed substantial evidence in support of overall apple (*BF*_10_ = 3.86) and rice preference change (*BF*_10_ = 25.57) as a function of the absence of shocks. A two-sided one-sample *t*-test showed a significant fall in apple and rice preference (for apple: *M* = −0.11, *SD* = 0.17, *t*(18) = −2.71, *p* = 0.014, 95% CI: [−0.19, −0.02], *d* = −0.62, and for rice: *M* = −0.08, *SD* = 0.09, *t*(18) = −3.73, *p* = 0.002, 95% CI: [−0.13, −0.04], *d* = −0.86).

Since both apple and rice showed significantly decreased preference over time, we looked at their correlations with increases in teak preference. We found that changes in apple and teak preference were correlated more compared to rice and teak (teak vs. apple: *r*(17) = −0.91, *p* < 0.001, 95% CI: [−0.96, −0.78]; teak vs. rice: *r*(17) = −0.68, *p* = 0.001, 95% CI: [−0.87, −0.33]). This suggests that in the absence of resource shocks, people preference shifted from the high-profit, proximal reward to the high-profit, delayed pay-off over time.

### Discussion

As the calculations illustrated in Figure 4 show, opting for the long-term plan (i.e., sowing teak) is the most profitable choice when resource shocks are minimal. Experiment 2’s participants appear to have discovered this fact inductively, as their teak preference grows and their apple and rice preference falls over time in the absence of intermittent, unpredictable resource shocks. Thus, consistent with the behavior of a utility maximizer, it seems that participants learned the nature of the payoffs over multiple game trials. This finding further confirms that the fall in long-term preference for teak, as seen in Experiment 1, was indeed brought on by the simulated experience of precarity and not by any other elements of the game’s design. Furthermore, this experiment provided empirical evidence for our model prediction that people with negligible resource shocks would still find the long-term horizon as their optimal planning duration.

## EXPERIMENT 3

Our first experiment showed that people shifted to a shorter planning horizon when confronted with multiple resource shocks, and in its absence, individuals could reasonably estimate the optimal choice and move toward a long-term planning horizon.

Now the question remains, what about the experience of precarity led to this contraction in time preference? We suspect that the unpredictability inherent in spurious resource demands leads to increased shifts in preference compared to conditions when resource demands are frequent yet predictable. We think that while functioning under low resources, people encountering unpredictable resource shocks would show lowered preference for the long-term option than people facing similar resource constraints and predictable resource shocks. Thus, we hypothesize that if unpredictability were primarily responsible for the shift in time preferences, we would observe a significant difference in teak preference between these conditions.

Experiment 3 investigates our hypothesis using a between-subject design where participants function under constrained resources; however, the predictability of the incoming resource shocks varied. The first arm of the experiment was identical to Experiment 1, in which people functioned under constrained budget and were unpredictably exposed to resource shocks. In the second arm, participants faced constrained resources similar to the first arm; however, resource shocks were made predictable by informing the participants of their impending arrival. We assessed how people’s crop preferences shifted in both conditions.

### Methods

#### Design Changes from Experiment 1.

To test our hypothesis, we implemented some changes in the paradigm, which are listed below:

▪  Change in the block structure - In the first experiment, the experimental paradigm had four randomized LV and HV blocks (two of each) after the practice block. Here, we started the experiment with the practice block, then one LV block, and lastly, two consecutive HV blocks. The two consecutive HV blocks created one extensive HV block. We limited the LV block to one, as resource shocks were not observed in LV blocks, making them ineffectual for shock-based computation of preference shifts. Thus, participants faced the same number of HV trials and half of the LV trials as experiment 1.
▪  Pre-sampled ordering of resource shocks - In experiment 1, the real-time sampled overrun could occur on any trial during the gameplay. In this experiment, however, we had pre-sampled the resource cost debits before the game, and all participants faced the same resource debits from their budgets.
▪  Number of overruns in HV block - Since, the resource debits was sampled in real-time in the first experiment, each participant faced a variable number of resource overruns (average being 10). Now, the HV block was designed such that resource debits would exceed budget one third of the time and had 48 trials in total. Thus, we kept the overruns faced by each participant constant at 16 in this experiment. This provided additional data points for our treatment and permitted us to examine the robustness of our resource shock hypothesis.
▪  Unpredictable and predictable resource shocks - In the first condition of this experiment, the resource cost debits were randomly dispersed during the HV block such that people could not predict when the resource shocks would happen. In the second ‘predictable’ condition, however, it was regularly timed, i.e., each resource shock occurred every six trials in the HV block, giving participants a sense of predictability of the turbulent times.
▪  Participants’ instructions - In this experiment, we asked people to do a ‘comprehension check’ after they read the game instructions, where they answered questions about the game environment and its controls. If unsure about any question or answered incorrectly, they were asked to return to the instruction sheet and answer again. This was done to ensure that people understood the instructions clearly. All pre- and post-experiment questionnaires were designed and implemented using PsyToolkit (Stoet, 2010, 2017). Lastly, to make the resource shocks predictable, participants were informed that after the initial practice block, they would experience some periods of stability, followed by extensive periods of turbulence, during which they would see increased resource cost deductions, land rent, and crop loss which is notified as “Crops are dying!” in-game. People were again notified of this at the start of the HV block.

#### Participants.

Using a Fixed-N BFDA (Bayes Factor Design Analysis) design with default prior Cauchy (0, sqrt(2)/2), an expected effect size of 0.25 (from the first experiment), a sample size *n* = 30 and a boundary BF (*BF*_*thres*_) = 3, we found the false positive and false negative evidence rates to be 0.021 and 0.26 respectively (Stefan et al., 2019). These estimated values are close to the general NHST criteria of accepted alpha and beta. We decided to start off with *n*_*min*_ = 30 in each condition, and compute *BF*_10_ after every participant until the *BF*_*exp*_ > = 3 (Schönbrodt et al., 2017). Priors used in BF calculation were the default settings of JASP (JASP Team, 2022).

Similar to the previous experiments, all participants were recruited via an online advert on campus. Since the experiment took around forty-five to sixty minutes to be completed, participants were compensated with $8 (PPP adjusted) for their time. In addition to this, three participants were also awarded bonus payment of $25, $15 and $5 (PPP adjusted) based on their performance in the game. The institutional ethics committee also approved this study.

### Results

We found that our data collection criteria were met with 30 participants as the computed bayes factor was found to be to be greater than the threshold (*BF*_*10*_ = 12.88 for teak difference between Unpredictable and Predictable condition > *BF*_*thresh*_ = 3, with default prior $r=\sqrt{2}/2$). Similar to last two experiments, preference deviations were calculated without the practice block and last six trials for each participant. With 1.75*IQR outlier exclusion criteria, we excluded two participants in the first group and none in the second^5^. Hence, the final data analysis is done with 28 (7 females, *M*_*age*_ = 21 years) and 30 participants (6 females, *M*_*age*_ = 22 years) in the first and the second group, respectively. (Supplementary analysis with alternate specifications and including the outliers can be found in Supplementary Information).

#### Crop Preference Shifts Across Conditions.

We followed the same analysis protocol in this experiment as in the first one: We derived the mean change in crop preference following resource shocks across participants in both groups and compared them as a metric of how time preference shifted as a function of predictability.

**Figure 8.**
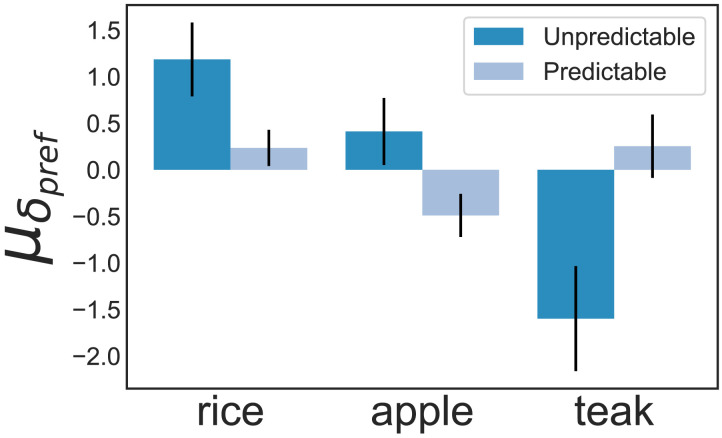
Results of Experiment 3: This diagram shows the mean shift in crop preference induced by resource shocks as found in ‘Unpredictable’ and ‘Predictable’ conditions. Teak preference shifts show statistical significance only in the ‘Unpredictable’ condition. The mean change in crop preference across all participants is denoted by *μ_δ_pref__*, and error bars signify standard error. *μ_δ_rice__, μ_δ_apple__*, and *μ_δ_teak__* for the ‘Unpredictable’ condition were found to be 1.19, 0.41, and −1.6, respectively. Similarly, *μ_δ_rice__, μ_δ_apple__*, and *μ_δ_teak__* for the ‘Predictable’ condition were 0.24, −0.49, and 0.25, respectively.

Since, the Brown-Forsythe test of equality of variance for teak preference shift showed significant heteroskedasticity (*F* = 4.87, *p* = 0.03), and we have unequal sample sizes for the two groups, we carried forward with Welch’s t test. For teak preference change, we hypothesized the shift to be higher in magnitude in the unpredictable case than the predictable one. We did a two-sided test for apple and rice preference shifts since we did not hypothesize a directional claim. Welch’s *t*-tests revealed that decreases in teak preference were higher in magnitude in the Unpredictable condition (*M* = −1.6, *SD* = 0.56) compared to the Predictable condition (*M* = 0.25, *SD* = 0.34), and it was statistically significant (*t*(44.69) = −2.81, *p* = 0.004, *r* = 0.27, 95% CI [−2.96, −0.75], *d* = 0.75, *BF*_10_ = 12.88). Preference for short-term crops significantly increased in the Unpredictable condition, i.e., mean change in preference for rice and apple increased in the presence of unpredictable shocks compared to predictable ones (Rice: *M*_*unpredictable*_ = 1.19, *SD*_*unpredictable*_ = 2.06, and *M*_*predictable*_ = 0.24, *SD*_*predictable*_ = 1.05, *t*(39.4) = 2.15, *p* = 0.04, 95% CI [0.06, 1.84], *d* = 0.58, *BF*_10_ = 1.78; and Apple: *M*_*unpredictable*_ = 0.41, *SD*_*unpredictable*_ = 1.87, and *M*_*predictable*_ = −0.49, *SD*_*predictable*_ = 1.25, *t*(46.5) = 2.11, *p* = 0.04, 95% CI [0.04, 1.76], *d* = 0.56, *BF*_10_ = 1.65). These results suggest that participants decreased their preference for the long-term option and compensatorily increased their preference for the short-term choices in the presence of unpredictable resource shocks compared to when shocks were predictable, as shown in Figure 8.

We also asked people to rate, on a scale of 0 to 10, how uncertain they felt when they saw “Crops are dying!” (0 being ‘not at all’ and 10 being ‘extremely’) on both conditions. We found the average ratings for the unpredictable and predictable conditions to be 7.5 and 5.5, respectively. This indicated that our experimental manipulation was successful and suggests that unpredictability is a factor of interest. Next, we ran post hoc tests on each condition, comparing the mean change in crop preference as a function of predictable and unpredictable resource shocks against the null change.

#### Crop Preference Shifts in ‘Unpredictable’ Condition.

**Figure 9.**
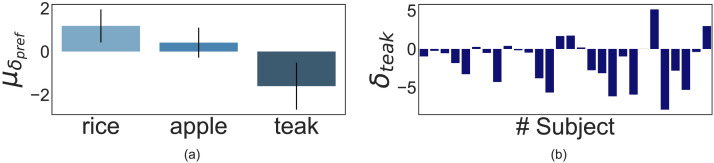
Results of ‘Unpredictable’ Condition: Panel **(a)** illustrates the mean shift in crop preference (*μ_δ_pref__*) across all participants. *μ_δ_rice__, μ_δ_apple__*, and *μ_δ_teak__* were found to be 1.19, 0.41, and −1.6, respectively. Error bars signify 95% CI. Panel **(b)** shows the teak preference shift (*δ_teak_*) data for all participants (*n* = 28). 20 participants showed negative teak preference post unpredictable resource shock.

For the Unpredictable condition, we see a similar trend as the first experiment—teak preference showed a significant negative change as a function of resource shocks (*M* = −1.6, *SD* = 2.93, *t(27)* = −2.83, *p* = 0.004, 95% CI [−2.54, −0.65], *d* = 0.54, *BF*_10_ = 10.44) as shown in Figure 9(a). Thus, this study further confirmed the result of the first experiment—people’s temporal planning horizon does contract due to multiple unforeseen resource overruns.

We found that preference shifts in apple and rice were both significantly correlated to changes in teak production post unpredictable shocks (apple vs. teak: *r(26)* = −0.72, *p* < 0.001, 95% CI [−0.86, −0.47]; and rice vs. teak: *r(26)* = −0.77, *p* < 0.001, 95% CI [−0.89, −0.56]). However, only rice preference showed a significant increase (*M* = 1.19, *SD* = 2.06, *t(27)* = 2.99, *p* = 0.006, 95% CI [0.37, 2.0], *d* = 0.565, *BF*_10_ = 7.25) and apple preference showed no significant change following shocks (*M* = 0.42, *SD* = 1.87, *t(27)* = 1.14, *p* = 0.26, 95% CI [[−0.33, 1.15], *d* = 0.22, *BF*_10_ = 0.36).

These findings suggest that people preferred the low-risk, short-term option more compared to the high-risk, short-term choice when they moved away from the long-term bet. This is at odds with the finding in experiment 1, where people preferred the high-risk, short-term crop choice more when their preference for the long-term crop decreased. We think people may have switched their risk preference in this experiment because they faced more resource overruns and choosing apples led to higher crop loss (and lowered net profit) than rice when frequency of shocks increased.

#### Crop Preference Shifts in ‘Predictable’ Condition.

**Figure 10.**
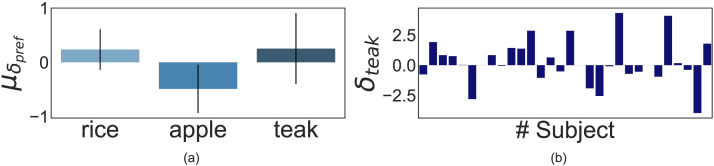
Results of ‘Predictable’ Condition: Panel **(a)** illustrates the mean shift in crop preference (*μ_δ_pref__*) across all participants. The mean shift in rice, apple, and teak preference is 0.24, −0.49, and 0.25, respectively. Error bars signify 95% CI. Panel **(b)** shows the within-participant teak preference shift (*δ_teak_*) data for all participants (*n* = 30). 13 participants showed negative teak preference post predictable resource shock.

Post hoc tests on the predictable condition showed that teak preferences did not change post resource shocks (*M* = 0.25, *SD* = 1.83, *t(29)* = 0.75, *p* = 0.77, 95% CI [−0.32, 0.83], *d* = 0.14, *BF*_10_ = 0.5) as shown in Figure 10(a). This suggests that participants’ preference for long-term crop choice did not fall in the presence of predictable resource overruns, even though they faced the same number of resource shocks as the previous condition.

Among the short-term crops, we found that rice showed no significant changes (*M* = 0.24, *SD* = 1.05, *t(29)* = 1.214, *p* = 0.235, 95% CI [−0.16, 0.63], *d* = 0.22, *BF*_10_ = 0.38) while apple showed a significant negative shift in preference post predictable shocks (*M* = −0.49, *SD* = 1.25, *t(29)* = −2.12, *p* = 0.04, 95% CI [−0.96, −0.02], *d* = 0.39, *BF*_10_ = 1.35) as shown in Figure 10(a). These results suggest that while people’s preference for long-term crops did not change, their preference for risky short-term crops fell in the presence of predictable overruns. This finding aligns with our previous intuition that people lowered their preference for the risky short-term crop because choosing more apples in the presence of frequent shocks would lead to higher crop loss and lower net profit.

Overall, comparing the effects of predictable and unpredictable shocks on long-term preference shows that a negative shift in teak preference is observable only under unpredictable shocks. Thus, present-centeredness was induced by the unpredictability brought on by resource shocks. Anticipated resource shocks, of similar frequency, was insufficient to produce deviations in long-term preference.

### Discussion

Experiment 3 replicates the results of experiment 1—when faced with resource shocks, people shift their planning horizon from a longer temporal scale to a shorter one. In our quest to find what is unique about the precarious environment, we found unpredictability to be the answer.

In the unpredictable condition, participants faced 16 unpredictable overruns each, and the effect size was 125% larger (Cohen’s d increased from −0.24 to −0.54) than in experiment 1, where participants faced an average of 10 overruns. In the predictable condition, participants again faced 16 resource shocks, but they showed no significant change in long-term preferences and the effect size decreased compared to the experiment 1 (Cohen’s d decreased from −0.24 to −0.14). Thus, our third experiment revealed that when individuals were confronted with more unpredictable resource shocks compared to the first experiment, the extent to which their temporal horizon contracted also increased significantly, and died down when shocks were made predictable. This demonstrates the robustness of our resource shock hypothesis—that the lived experience of precarity leads to reduced time preference—and demonstrates that the uncertainty associated with precarity considerably dominates this effect.

Among the short-term crop choices—we found that participants favored the low-profit, low-risk rice more in experiment 3 compared to the high-profit, high-risk apple in experiment 1 when switching their preference from the long-term crop choice. We think that this switch in risk preference can be understood in light of the optimal crop choice calculation, which shows that as the probability of resource shocks increase, choosing apple leads to increased crop loss and lowered net crop profit compared to rice. While our sample in this experiment encountered 16 resource shocks per game, participants in experiment 1 encountered 10 shocks on average. Since apple was the high-risk crop choice, increased shocks would lead to higher cumulative crop loss and lower crop yield than rice. Participants may have inductively arrived at this understanding because of the extensive HV block (as observed in the negative shift of apple preference in the predictable condition), which made them prefer the low-risk short-term crop option more in experiment 3.

## GENERAL DISCUSSION

Financial instability as a critical determinant in sustaining the poverty trap is a well-studied topic (Gennetian & Shafir, 2015). Earlier literature shows evidence of negative income shocks causing decreased valuation of future monetary options (Bickel et al., 2016), fast foods (Mellis et al., 2018), and even devaluing future options and being present-biased when controlling for income levels between rich and poorly endowed participants (Haushofer & Fehr, 2013). However, studies showing changes in time preference have primarily been conducted using one-shot, static paradigms like a delay discounting questionnaire. Our study improves previous paradigms by presenting participants with a dynamic game environment with intermittent periods of stability and volatility and measuring the real-time evolution of people’s time preferences.

In this paper, we set out to test whether functioning under a limited budget with random resource shocks could induce temporal discounting in people. While existing literature investigating the causes of present-bias behavior has little to say as to *how* financial scarcity leads to future discounting, our hypothesis places the mechanism front and center. We argue using simulations that environmental uncertainty as random, frequent exogenous shocks deplete budgets fast, causing future plans to fail. Using a series of three experiments, we show that observers experiencing unpredictable resource shocks switch their preferences to more present-centric options.

Furthermore, using our dynamic game environment, we were able to identify the critical role of unpredictable resource demands on temporal preferences. The existing ‘scarcity’ literature places the scarcity of essential resources (money or time) as the primary cause of present-centric behavior in individuals living in low SES communities. In Mani et al. (2013)’s study, low income individuals performed worse on cognitive tests when exposed to sudden, substantial expenses. This led the authors to suggest that lack of money impedes cognitive functioning and leads to sub-optimal choices. Our results add to the existing literature by highlighting unpredictable environmental demands as a critical variable of interest whose effects are exacerbated by the scarcity of available resources. Thus, while lack of resources may be crucial, we think the incidence of substantial unexpected expenses leads to make present-focussed choices. In other words, while lack of resources may be a necessary condition to preference shifts, our results suggest that resource scarcity alone is not sufficient to explain present biased behavior.

Based on our ‘resource shock’ hypothesis, we hypothesized that in a perfectly predictable world, having low resources would not affect people’s temporal choices, and they would plan for the future. We found this to be the case in our second experiment where people’s preference for the long-term plan increased under conditions of budgetary constraints and null turbulence. However, an *unpredictable* world filled with over-the-budget resource demands forces people to be present-biased. This was evident in our first and third experiments where lab-simulated environmental precarity led to shifts in temporal preference, aligned with experience effects seen in observational field studies (Malmendier, 2021b; Malmendier & Nagel, 2011). Thus, uncertainty seems to be sufficient to induce present-centric behavior in our experiments. However, we think that smaller endowments would amplify the burden of unpredictable shocks, thereby making them more fatal. Thus, resource constraints coupled with unpredictable turbulence appear to be necessary and sufficient for individuals to be present-focussed.

Even though long-term planning is often construed as the rational choice, we show via simulations that moving to a shorter planning horizon is rational when one has low budgetary endowments and faces recurrent, unforeseen resource demands. This suggests that optimality is malleable and dynamic under environmental constraints, and rationality is contingent upon the ecological conditions one encounters. In other words, people’s inter-temporal preferences are sensitive to the perturbations encountered in their environment, and preferences wax and wane based on these ecological constraints. Thus, our results align well with the ‘ecological rationality’ hypothesis (Todd & Gigerenzer, 2012) of temporal discounting and the ‘resource rational’ analysis (Bhui et al., 2021), which conceives context-dependent optimality arising from the interactions of the individual and their environment and explains present-centeredness as a rational adaptation to ecological constraints.

We conclude by observing that identifying the correct mechanism mediating the interaction between structural factors and behavior is particularly crucial for designing efficient poverty alleviation and other welfare policies. Our work suggests a necessity to design interventions that predictably buffer people against unexpected planning failures in addition to economic considerations. Moreover, uncertainty can exacerbate a perceived lack of control over one’s decisions and outcome (Pepper & Nettle, 2017), making them more pessimistic about future prospects, as often observed in people living in low socio-economic conditions (Das et al., 2020). Thus, welfare interventions such as cash transfers or free healthcare are expected, from this perspective, to not only support the already-constrained budgets of people with low income but also buffer them mentally against psychological forces brought on by precarity. In contrast, other interventions like debt relief or access to micro-credit loans may relieve economic constraints temporarily but would not remove the mental constraints produced by having to guard against unexpected expenses. Thus, interventions that can predictably buffer against precarious environmental conditions are likely to change present-centricity in individuals living in poverty.

## ACKNOWLEDGMENTS

We are grateful to our participants, colleagues, and staff, who enabled us to carry out the experiment. Also, a special mention should go to the subreddit r/gamemaker and youtube channel FriendlyCosmonaut for their help in designing the game. We would also like to thank Revati Shivnekar for her help in debugging and testing the paradigm during its development.

## FUNDING INFORMATION

This study has been funded by contributions from the Centre of Behavioral and Cognitive Sciences, Allahabad and the Department of Cognitive Science, IIT Kanpur.

## AUTHOR CONTRIBUTIONS

Arjun Mitra: Data curation; Formal analysis; Investigation; Methodology; Software; Validation; Visualization; Writing – original draft. Narayanan Srinivasan: Conceptualization; Formal analysis; Methodology; Project administration; Resources; Supervision; Validation; Writing – review & editing. Nisheeth Srivastava: Conceptualization; Formal analysis; Funding acquisition; Methodology; Project administration; Resources; Supervision; Validation; Visualization; Writing – original draft; Writing – review & editing.

## DATA AVAILABILITY STATEMENT

All materials, code, and data can be found in the OSF Repository (https://osf.io/6uyma/?view_only=abd4aca2e5a34492ab424bd8da8fdc34).

## Notes

^1^ To quantify the robustness of our simulations, we varied the distribution of costs and nature of reward increments and found identical results. For more details, please see Supplementary Information.^2^ Unless stated otherwise, we imply the word ‘preference’ to mean peoples’ revealed preferences throughout this paper.^3^ The last six trials were excluded from crop deviation calculation, as participants noted that they were motivated to not choose the long-term crop at the end of the game. Thus, if a resource shock occurred on these boundary conditions, we dropped those trials from analysis.^4^ With these specifications, we obtained the sample size and the probability of *BF*_01_ being greater than 3 given *H*_0_ is true or *BF*_10_ being greater than 3 given *H*_1_ is true. For the sample size of 180 participants, the probability of *BF*_01_ > 3 and *BF*_10_ > 3 was calculated to be 0.94 and 0.80, respectively.^5^ We used a 1.75*IQR outlier calculation in all experiments instead of a 1.5*IQR criterion to make the data exclusion criteria more stringent.
